# Dosimetric comparison of three intensity‐modulated radiation therapies for left breast cancer after breast‐conserving surgery

**DOI:** 10.1002/acm2.12287

**Published:** 2018-03-10

**Authors:** Huai‐wen Zhang, Bo Hu, Chen Xie, Yun‐lai Wang

**Affiliations:** ^1^ Department of Radiotherapy Jiang‐Xi Cancer Hospital Nanchang China; ^2^ Key Laboratory of Nondestructive Testing (Ministry of Education) Nanchang Hang Kong University Nanchang China; ^3^ Department of Radiation Oncology PLA General Hospital Beijing China

**Keywords:** breast cancer after conserving surgery, dosimetry, field‐in‐field (FIF)–direct machine parameter optimization, intensity‐modulated radiation therapy, radiation therapy

## Abstract

**Purpose:**

This study aimed to evaluate dosimetric differences of intensity‐modulated radiation therapy (IMRT) in target and normal tissues after breast‐conserving surgery.

**Methods:**

IMRT five‐field plan I, IMRT six‐field plan II, and field‐in‐field–direct machine parameter optimization–IMRT plan III were designed for each of the 50 patients. One‐way analysis of variance was performed to compare differences, and *P* < 0.05 was considered statistically significant.

**Results:**

Homogeneity index of plan III is lower than those of plans I and II. No difference was identified in conformity index of targets. Plan I exhibited difference in mean dose (*D*_mean_) for the heart (*P* < 0.05). Plan I featured smaller irradiation dose volumes in *V*_5_, *V*_20_ (*P* < 0.05) of the left lung than II. Plan I exhibited significantly higher *V*_5_ in the right lung than plans II and III (*P* < 0.05). Under plan I, irradiation dose at *V*_5_ in the right breast is higher than that in plans II and III. Patients in plan III presented less total monitor unit and total treatment time than those in plans I and II (*P* < 0.05).

**Conclusion:**

IMRT six‐field plans II, and field‐in‐field–direct machine parameter optimization–IMRT plans III can reduce doses and volumes to the lungs and heart better while maintaining satisfying conformity index and homogeneity index of target. Nevertheless, plan II neglects target movements caused by respiration. In the same manner, plan III can substantially reduce MU and shorten patient treatment time. Therefore, plan III, which considers target movement caused by respiration, is a more practical radiation mode.

## 1 INTRODUCTION

Breast cancer is one of the most common malignant tumors in females.^1^, ^2^ The population of breast cancer patients in China reached an annual growth rate of approximately 3% to 4% in the past years.^3^ Most patients with breast cancer can receive operative treatment in the early stage with continuous increase in medical technologies and quality. Breast is a secondary sex characteristic of women, whereas esthetics has become an important treatment requirement of patients with breast cancer owing to increasing attention on esthetic effect and living quality.^4^ Therefore, comprehensive therapy of chemoradiotherapy after breast‐conserving surgery of breast cancer has become the standard treatment for patients with early breast cancer.^5^ This therapy has also achieved curative effects similar to those of radical mastectomy. Different patients exhibit significant differences in breast shapes and widths at different parts of the breast. The common clinical two‐side tangent field radiotherapy exhibits an uneven irradiation dose in the target region and high irradiation doses to key organs, such as the heart and lungs. Fixed‐field intensity‐modulated radiation therapy (IMRT) shows significant advantages in improving irradiation dose distribution in the target region and reducing irradiation dose in surrounding normal tissues.^6^, ^7^, ^8^ However, IMRT expands low‐volume irradiation region of normal tissues and increases irradiation doses to the lungs and breasts at the healthy side.^9^, ^10^, ^11^, ^12^ In this study, IMRT fixed five‐field plan, IMRT fixed six‐field plan, and field‐in‐field (FIF)–direct machine parameter optimization (DMPO)–IMRT plan were designed for different patients according to the target region using the Pinnacle^3^ (Philips Medical System, Andover, MA, USA) treatment plan system version 9.6. Dosimetric comparison was conducted on dose distribution, conformity index (CI), and homogeneity index (HI) of the target region, and irradiation dose in the heart, lungs, and healthy breast of the three IMRT plans. The present study aimed to provide references for clinical treatment after breast‐conserving surgery of early breast cancer.

## 2 MATERIALS AND METHODS

### 2.A Patients

A total of 50 patients who received breast‐conserving surgery of the left early breast cancer were admitted in the radiotherapy department of Jiang‐Xi Cancer Hospital from January 2012 to December 2016. All patients received left breast lumpectomy and axillary lymph node dissection. Surgical margin was negative according to pathological diagnosis. The patients exhibited neither blood vessel invasion nor lymphatic metastasis in axillary lymph node dissection. Patients presented no history of heart and respiratory system diseases. The upper limbs were exercised fully after surgery, meeting postural therapy. Radiotherapy was implemented after complete union of surgical incision. No hydrops in the wound were observed. Patients showed normal cardiopulmonary function and no radiotherapy contraindication.

Informed consent forms were signed by all patients. The study was performed in accordance with the Declaration of Helsinki and was approved by the Ethics Committee of Jiang‐Xi Cancer Hospital.

### 2.B Posture fixation and computed tomography scanning

Patients lay on the Varian Acuity digital analog machine. The left arm was raised upward at 90° and fixed by a Med‐Tec 250 breast bracket. The breast with cancer was exposed completely. Corresponding reference points were marked by laser positioning. Scar wire was used to mark around the anterior median line, mid‐axillary line, first anterior rib level, and 2 cm below the breast wrinkle. SOMATOM Definition AS 20 spiral computed tomography (CT) was used to scan from the supraclavicular region to 5 cm below the breast wrinkle under free‐breathing state. Normal tissues and organs surrounding the target region, such as the lung, heart, liver, and contralateral breast, were covered completely. Scan thickness totaled 5 mm. CT images were transmitted to the Pinnacle^3^ treatment planning system version 9.6 through radiotherapy of special local area networks.

### 2.C Planning target volumes and organs at risk

**Figure 1:**
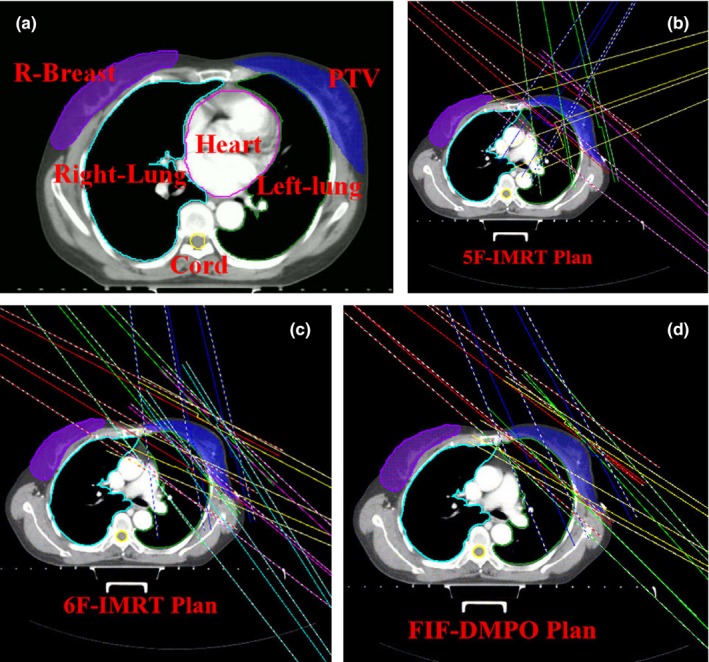
(a) Example of the contour of PTV and OARs; (b), (c), and (d) show the beam arrangement of the three radiotherapy techniques.

A 3D model was reconstructed based on CT images of patients with breast cancer in 3D treatment planning software. Radiotherapy target regions and organs at risk (OARs) of all patients were sketched by the same physician at the doctor workstation of the Pinnacle^3^ treatment planning system version 9.6 according to the 50th^13^ and 62nd^14^ reports of the International Commission on Radiation Units and Measurements (ICRU) to avoid personal error. Clinical target volume (CTV) is defined as the complete area of mammary tissues, inter‐pectoral lymph nodes, and lymphatic drainage at the chest wall. The front boundary lies 0.5 cm below the skin surface. Planning target volume (PTV) was used to expand the internal and external boundaries outward by 0.8 cm and expand the upper and lower boundaries by 1.2 cm based on CTV. The front boundary is similar to that of CTV. Rear boundary was expanded outward by 0.5 cm. Lung tissues were excluded from PTV. Simultaneously, OARs were defined; these organs included the lungs at two sides, heart, spinal cord, and contralateral breast (Fig. 1(a)).

### 2.D Treatment planning

A radiation therapy plan was generated to deliver ideal dose distribution, which has been determined by the radiotherapist, to the target. Three different IMRT plans were designed for each patient using the 6 MV photon beams of a Precise linear accelerator (Elekta AB, Stockholm, Sweden). Step‐and‐shoot beams were used for the three IMRT plans. Dose calculations in all three plans were performed using the collapsed‐cone convolution algorithm with heterogeneous corrections on a dose grid with 3.0 × 3.0 × 3.0 mm^3^ resolution. The patients were treated with postoperative radiotherapy to a prescribed dose of 5000 cGy in 200 cGy fractions for 5 days per week. All three plans require that 95% of PTV reaches the prescribed dose of 5000 cGy. On the other hand, dose limits of OARs were determined in all three plans: spinal cord: *D*_max_ < 3000 cGy; lung at the left side: *V*_20_ < 25% and mean dose (*D*_mean_) <1500 cGy; lung at the right side: *V*_5_ < 15% and *D*_max_ < 1000 cGy; the entire lung: *V*_20_ < 20%; heart: *V*_30_ < 10% and *V*_40_ < 5%; right breast: *D*_max_ < 1000 cGy and *D*_mean_ < 800 cGy. All three plans use the same optimization objective. Optimization prioritized normal tissue constraints. Figs. 1(b), 1(c), and 1(d) show the beam arrangement of the three radiotherapy techniques.

1. IMRT five‐field plan (plan I): Five radiation fields were designed. The direction of conventional tangent field was used as the start–stop incidence direction. Incident angles of all radiation fields were equal in the plane at the breast with cancer. Radiation fields of IMRT have not been extended during the design. DMPO parameters of radiotherapy plan were set according to the dose of planning target volume and dose limits for OARs. Ideal dose distribution was achieved by repeated adjustment and optimization of parameters.
2. IMRT six‐field plan (plan II): Rack angular distribution was approximately tangent to the lung edges. Radiation fields were distributed clockwise. The first, second, and third radiation fields were staggered by approximately 10° to 20° and were located at the upper part of PTV. By contrast, the fourth, fifth, and sixth radiation fields were staggered by approximately 10° to 20° and were located at the lower part of PTV. The other planning parameters were set similar to those in plan I.
3. FIF‐DMPO‐IMRT plan (plan III): The total prescribed dose in PTV (5000 cGy) was divided into two parts, namely, 3500 cGy to 4000 cGy of FIF irradiation dose and 1000 cGy to 1500 cGy of IMRT irradiation dose. These values indicate that each of the prescribed doses of radiation (200 cGy) was divided into two parts, namely, 140 cGy to 160 cGy of FIF irradiation dose and 40 cGy to 60 cGy of IMRT irradiation dose. (a) FIF irradiation dose totaling 3500 cGy to 4000 cGy: Two tangent directions of the target region were used as incidence direction of the main field of FIF radiation. First, the target region was extended uniformly outward by 0.5 cm after removal of the wedge‐shaped filtering plate from the conventional tangent field. Second, multileaf collimator (MLC) in the breast target region close to the radiation field at the airside extended toward the skin surface by 1.5 cm. This phenomenon prevents radiation leakage, which is caused by respiratory movement, in the target region. MLC close to the lung tissue shrank by 0.2 cm to reduce irradiation volume to lung tissues as much as possible. Third, isodose weights from the internal field to the external field were calculated. Subfields were set on the directions of internal and external tangent fields at different levels of high‐volume regions. High dose in the breast target region was shielded by MLC level by level to gradually narrow the high‐volume regions in subfields and prevent further production of these areas. By contrast, regions with inadequate dose in the target region were supplemented by one to two subfields. Thus, relatively uniform dose distribution in the breast target region was obtained. (b) IMRT irradiation dose of 1000 cGy to 1500 cGy: One to two fields, which deviated from the rack angle in step II by approximately 5° to 10°, were set at the upper and lower parts of PTV. IMRT irradiation dose was optimized through the minimum number of radiation subfields, minimum subfield area, irradiation dose, and iterations under opening FIF irradiation dose. This condition can increase HI of the target region and reduce complications to normal tissues.

### 2.E Plan analysis and evaluation

Three IMRT plans were compared in terms of HI and CI of the target region and volume dose of related normal tissues by dose–volume histogram (DVH). Evaluation parameters of PTV included the following: (1) *D*_mean_, maximum dose (*D*_max_), and minimum dose (*D*_min_) of PTV. (2) The HI was defined as follows^15^: HI = D_5_/D_95_, where D_5_ is prescribed dose to cover 5% of PTV, and D_95_ is prescribed dose to cover 95% of PTV. HI reflects the uniformity of doses in the target region. A high HI indicates poor uniformity of dose distribution in the target region. (3) The CI was used to evaluate the conformity degree of the target region and reference iso‐dose surface. Calculation formula of CI^16^ is as follows: CI = (*V*_t,ref_/*V*_t_) × (*V*_t,ref_/*V*_ref_), where *V*_t,ref_ refers to the PTV covered by 95% of the prescribed dose, *V*_t_ is the total PTV, and *V*_ref_ represents the total volume covered by 95% of the prescribed dose. CI ranges between 0 and 1. A high CI indicates good conformity degree. Evaluation parameters of OARs included the following: left lung: *D*_mean_, *V*_5_, and *V*_20_; right lung: *D*_mean_ and *V*_5_; heart: *D*_mean_, *V*_30_, and *V*_40_; right breast: *D*_mean_ and *V*_5_; and spinal cord: *D*_max_, *D*_min_, and *D*_mean_. *V*_*x*_ reflects the proportion of volume under *x* Gy radiation in total volume.

### 2.F Statistical method

All DVH data were inputted into and analyzed by SPSS 17.0. Quantitative data were expressed as mean ± standard deviation ($\bar{x}\pm s$). Differences in the three IMRT plans were compared by one‐way analysis of variance (ANOVA). Further pair‐wise comparison was conducted to determine statistical significance. Data conforming to normal distribution were investigated by ANOVA. The remaining data were analyzed by nonparametric rank and summing test. *P* < 0.05 represents statistically significant difference.

## 3 RESULTS

**Figure 2:**
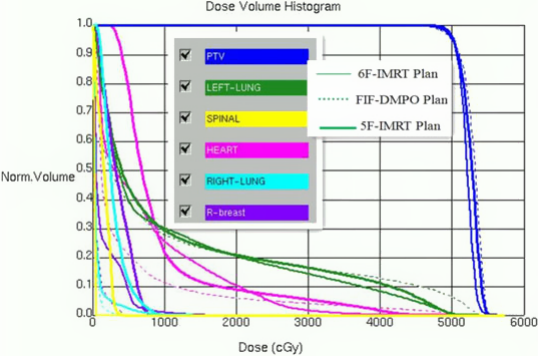
DVHs of three treatment plans for one representative patient.

Figure 2 shows the corresponding DVHs in the three treatment plans for one representative patient.

### 3.A Comparison of PTV, HI, and CI

Table 1 summarizes PTV _(max, min, mean)_, HI, and CI of the target region in plans I, II, and III. Under the premise that 95% iso‐dose curve covers PTV, plan I exhibits a higher *D*_min_ of PTV than the other two plans; plan II exhibits a slightly small mean dose of PTV; and plan III features a slightly poor HI. These three IMRT plans exhibit no statistically significant difference in *D*_max_ of PTV and CI of the target region (*P* > 0.05).

**Table 1:** Comparison of PTV dose, CI, and HI among the three IMRT plans *x *±* s*

Project indicators	Plan I	Plan II	Plan III
PTV_mean_(cGy)	5242.24 ± 28.08	5232.60 ± 29.06^a^, ^b^	5278.18 ± 20.91
PTV_max_(cGy)	5584.62 ± 25.23	5593.20 ± 44.00	5606.34 ± 40.56
PTV_min_(cGy)	4250.14 ± 261.38^a^	3958.20 ± 465.14	4054.16 ± 170.67
HI	1.08 ± 0.01^a^	1.07 ± 0.01^a^	1.09 ± 0.00
CI	0.81 ± 0.10	0.77 ± 0.11	0.75 ± 0.09

a Compared with plan III, *t *=* *3.516, 4.008, 3.551, and 4.811 (*P* < 0.05).

b Compared with plan I, *t *=* *3.551 (*P* < 0.05).

### 3.B Comparison of irradiation dose and volumes of OARs under the three IMRT plans

Table 2 lists the irradiation doses and volumes of the three IMRT plans in the heart tissues, left and right lungs, right breast, and spinal cord.

**Table 2:** Irradiation doses and volumes in the OARs of the three IMRT plans (%) *x *±* s*

Organ	Project indicators	Plan I	Plan II	Plan III
	*D*_mean_	1424.62 ± 268.65^a^	522.84 ± 137.17	431.66 ± 168.80
Heart	V30	4.55 ± 5.19	1.41 ± 0.98	4.30 ± 4.41
	V40	0.74 ± 0.71	0.23 ± 0.20	2.76 ± 3.19
	*D*_mean_	1150.30 ± 42.08^a^	1141.90 ± 84.64	1036.08 ± 85.31
Left lung	*V*_5_	43.67 ± 1.07	44.55 ± 4.11	35.02 ± 14.11
	*V*_20_	22.04 ± 0.85	20.59 ± 1.68	15.49 ± 8.23
	*D*_mean_	521.04 ± 161.88^a^	44.56 ± 15.29^b^	38.46 ± 16.59
Right lung	*V*_5_	51.12 ± 26.28^a^	0.04 ± 0.09b	0.47 ± 0.66
	*D*_mean_	458.23 ± 219.08^a^	108.88 ± 45.64	94.95 ± 74.47
Right breast	*V*_5_	44.92 ± 29.25^a^	2.80 ± 3.76^b^	1.62 ± 3.63
	*D*_max_	592.02 ± 193.34^a^	68.92 ± 32.00^b^	37.50 ± 11.93
Spinal cord	*D*_min_	44.52 ± 72.19	8.84 ± 9.80	7.96 ± 4.18
	*D*_mean_	223.44 ± 101.27^a^	32.22 ± 12.00^a^, ^b^	19.00 ± 8.43

a Compared with plan III, *t *=* *4.186, 3.212, 6.854, 4.358, 3.411, 3.512, 6.654, 4.714, 3.061 (*P* < 0.05).

b Compared with plan III, *t *=* *6.130, 4.158, 3.194, 6.641, 4.742 (*P* < 0.05).

1. Dose and volume in the heart
  - Table 2 lists the irradiation doses and volumes of the three IMRT plans in the heart tissues (heart). Plan I yielded a significantly higher irradiation D_mean_ than plans II and III according to irradiation dose in the heart. The findings showed statistical difference (*P* < 0.05). The three IMRT plans exhibited no significant difference in high‐level irradiation volumes (*V*_30_ and *V*_40_). These results indicate no statistical difference (*P* > 0.05).
2. Irradiation dose and volume in the left lung
  - Table 2 displays the irradiation doses and volumes in the left lung of the three IMRT plans (left lung). *D*_mean_ in the left lung of plan I is slightly higher than those of plans II and III. However, no statistically significant difference was observed between plans II and III. No statistically significant difference was also observed among the three IMRT plans in terms of *V*_5_ and *V*_20_. All three IMRT plans can meet clinical requirements of irradiation dose and volume limits to the left lung.
3. Irradiation dose and volume in the right lung
  - Table 2 summarizes the irradiation doses and volumes in the right lung of the three IMRT plans (right lung). Irradiation doses (*D*_mean_) in the right lung of plan I are significantly higher than those of plans II and III. Results showed statistical difference (*P* < 0.05). The difference between plans II and III exhibited no statistical significance (*P* > 0.05). *V*_5_ in the right lung of plan I is significantly higher than those of plans II and III. Plan I contains five average fields in the hemisphere. This phenomenon increases irradiation errors to tissues at the healthy right lung to some extent. This result also explains the higher *V*_5_ of plan I than those of plans II and III (*P* < 0.05). Therefore, field settings of plans II and III show absolute advantages with respect to protection of the healthy right lung.
4. Irradiation dose and volume in the right breast
  - Table 2 provides the irradiation doses and volumes in the right breast of the three IMRT plans (right breast). Plan I showed higher *D*_mean_ dose in the right breast than plan III, showing statistical difference (*P* < 0.05). Plans II and III exhibited no statistically difference (*P* > 0.05). Plan I exhibited higher *V*_5_ in the right breast than plans II and III. In the arrangement of tangent fields, plans II and III protected the right breast first. This condition resulted in lower irradiation dose in the right breast compared with plan I. All three plans featured small *D*_min_ in the right breast. These findings showed no statistical difference (*P* > 0.05).
5. Irradiation dose and volume in the spinal cord
  - Table 2 presents the irradiation doses and volumes in the spinal cord of the three IMRT plans (spinal cord). The three IMRT plans feature equivalent *D*_min_ in the spinal cord. These findings indicated no statistical difference (*P* > 0.05). However, plan I yielded higher *D*_max_ and *D*_mean_ than plans II and III, showing statistical difference (*P* < 0.05).

### 3.C Comparison of total monitor unit and total treatment time of patients under the three IMRT plans

Table 3 shows the total monitor unit and total treatment time of patients of the three IMRT plans. Total monitor unit and total treatment time of patients in plan III measured less than those of plans I and II (*P* < 0.05).

**Table 3:** Irradiation doses and volumes in the spinal cord of the three IMRT plans (%) *x *±* s*

Project indicators	I	II	III
Monitor unit (MU)	553.4 ± 109.90^a^	489.6 ± 129.00^b^	325.00 ± 44.72
Treatment time (Min)	5.52 ± 0.99^a^	5.60 ± 1.52^a^, ^b^	1.64 ± 0.16

a Compared with plan III, *t *=* *4.303, 8.655, and 0.059 (*P* < 0.05).

b Compared with plan III, *t *=* *2.696 and 5.770 (*P* < 0.05).

## 4 DISCUSSIONS

To date, radiotherapy after breast‐conserving surgery has been the standard treatment for early breast cancer. Adjuvant chemoradiotherapy after breast‐conserving surgery cannot only reduce local recurrence risk effectively but also decrease distant metastasis rate and increase survival rate and quality of life of patients significantly.^17^, ^18^ Thus, the same curative effect with radial operation or modified radical operation is achieved. However, the approximate hemisphere of the breast determines significant differences in source–skin distance at different parts.^19^, ^20^ This factor will cause poor HI and CI in the target region after breast‐conserving surgery and skin ulcer, radiation pneumonitis, and cardiac trauma. Therefore, current research focuses on means for further minimizing irradiation doses/volumes of OARs and improving dose homogeneity of targets.^7^

In this study, dosimetric comparison of three IMRT plans was conducted from the target region and surrounding important organs of adjuvant chemoradiotherapy after left breast‐conserving surgery. All these plans can achieve good dose coverage to the target region. Plan III possessed slightly lower HI than plans I and II because dose uniformity in the target region is positively correlated with the number of planning radiation fields and subfields. Plans I and II, which are full IMRT modes, have a significantly higher numbers of radiation fields and subfields than plan III.

Radiation pneumonitis and pulmonary fibrosis are important complications of radiation‐induced pneumonitis for breast cancer.^21^, ^22^, ^23^ Occurrence rate of complications is significantly correlated with irradiation volume and dose in lung tissues. *V*_5_ and *V*_10_ in lung tissues are important factors influencing occurrence of radiation pneumonitis.^24^, ^25^ Research has shown that occurrence rate of radiation pneumonitis reaches higher than 20% when *V*_10_ of the lung measures higher than 50%.^26^ *V*_5_ and *V*_10_ must be reduced as much as possible while controlling *V*_20_ to reduce occurrence rate of radiotherapy lung injury. No statistically significant difference was observed among the three IMRT plans in terms of *V*_5_ and *V*_20_ in the left lung. Plans II and III comprehensively consider the effect of radiation field direction on lung tissues. In these two plans, radiation fields deviate by 5° to 10° along the original tangent field. This result can increase irradiation dose in the target region and avoid excessive irradiation errors to lung tissues. Specifically, plan III reduces irradiation to lung tissues by shrinking MLC close to the lung tissue by 0.2 cm. The aim is to reduce irradiation volume to lung tissues. *D*_max_ in the left lung of plan III is significantly smaller than those of plans I and II. *D*_min_ of plan II is slightly smaller than that of plan III. *D*_mean_ in the left lung of plan I is significantly higher than those of plans II and III. However, no statistically significant difference was observed in *D*_mean_ in the left lung between plans II and III. All three IMRT plans can meet clinical irradiation volume limits in the left lung tissue. Irradiation dose in the right lung is mainly caused by X‐ray scattering and leakage between lung lobes. Plan I adopts a uniform field arrangement in the hemisphere, which cannot prevent irradiation to the right lung tissues. Therefore, *V*_5_ at the right lung in plan I is 51.12 ± 26.28. This value is significantly higher than those of plan II (0.04 ± 0.09) and plan III (0.47 ± 0.66). Plan I also achieved significantly higher *D*_mean_ in the right lung in comparison with plans II and III.

Radiotherapy‐induced heart diseases correspond to a group of clinical and pathological conditions of heart injuries caused by irradiation; these injuries include ventriculus sinister functional injury and pericardium injury. Research has shown a dose–effect relationship between occurrence of radiotherapy‐induced heart diseases and irradiation dose and volume in the heart.^27^ When irradiation dose in the heart is smaller than 3000 cGy, occurrence rate of radiotherapy‐induced heart diseases reduces significantly, whereas that of coronary ischemia caused by IMRT is low, and this result is related to the small *V*_30_.^28^ The three plans yielded a small *V*_30_ without significant differences and low *D*_mean_ in the heart. These results indicate that all three plans can protect the heart. Plan I presented significantly higher *D*_mean_ than plans II and III as it applies an incidence field that is approximately perpendicular to the breast and runs through the heart.

Low irradiation dose in the healthy breast is an important cause of right breast cancer after radiotherapy.^29^, ^30^ Gao et al. proved that healthy breast may suffer secondary cancer after radiotherapy of 2.9 Gy to 4.3 Gy.^31^ This phenomenon is caused by high irradiation dose in healthy breast tissues. Di Betta et al. advocated the use of 5 Gy irradiation dose as optimal irradiation dose to surrounding healthy tissues.^32^ Kaufman et al. declared that radiotherapy after surgery for breast cancer may increase the risk of lung cancer.^33^ In this study, plan I exhibited significantly higher *V*_5_ in the right breast than plans II and III. This result is attributed to the selection of radiation fields. This condition involves the risks of radiotherapy‐induced secondary cancer. *D*_mean_ in the right breast is smaller than 5 Gy in plans II and III. *V*_5_ values totaled 2.80 ± 3.76 and 1.62 ± 3.63, showing no statistical difference. Therefore, plans II and III can prevent primary cancer to the right healthy breast effectively.

Plans II and III are superior to plan I in reducing irradiation dose and volume in OARs (i.e., lungs, heart, and spinal cord) while maintaining CI and HI of the target region when the prescribed dose is 50 Gy as viewed from physical examination. Plans II and III have exhibited lower occurrence rate of complications than plan I. However, plan II uses full IMRT without considering the effect of target movement caused by respiration on dose distribution. Research has shown a maximum front–back error totaling 0.2–0.3 cm.^7^, ^34^ This value is related to breast attachment to external chest walls, large body movement, and significant changes in irradiation positions caused by different rising degrees of the arms. Respiratory movement of patients will cause significant movement of the target region.^35^, ^36^ All of these factors should be considered in IMRT design. Plan III based on the combination of FIF, which is dominated by the tangent field and DMPO‐IMRT, considers the influences of target region movement caused by respiration on actual irradiation dose distribution of patients. Thus, clinical CI and HI of the target region are satisfied. Field arrangement along the tangent direction can significantly reduce low‐volume regions in lung tissues, decreasing the probability of occurrence of radiation pneumonitis and radiation heart diseases. Therefore, the combination of FIF and DMPO‐IMRT is a practical method of radiotherapy after breast‐conserving surgery of left breast cancer.

## 5 CONCLUSIONS

This study aimed to evaluate the dosimetric differences in three IMRT in the target region and surrounding normal tissues after breast‐conserving surgery of early breast cancer. Compared with plan I, plans II and III can reduce dangerous irradiation doses and volumes to the lung, heart, and spinal cord better while maintaining satisfying CI and HI of the target region in clinical treatment. Nevertheless, plan II neglects the effect of target region movement caused by respiration on dose distribution. Therefore, plan III, which considers target region movement caused by respiration and combines FIF and DMPO‐IMRT serves as a more practical radiation mode after breast‐conserving surgery of breast cancer.

## COMPETING INTERESTS

The authors declare no competing financial interests.

## References

[1] Chen W , Zheng R , Zhang S , et al. Cancer incidence and mortality in China in 2013: An analysis based on urbanization level. Chinese J Cancer Res. 2017;29:1–10. 10.21147/j.issn.1000-9604.2017.01.01 PMC5348470 28373748

[2] Chen W , Zheng R , Baade PD , et al. Cancer statistics in China, 2015. CA Cancer J Clin. 2016;66:115–132. 26808342 10.3322/caac.21338

[3] Parkin DM , Bray F , Ferlay J , Pisani P . Global cancer statistics 2002. CA Cancer J Clin. 2005;55:74–108. 15761078 10.3322/canjclin.55.2.74

[4] Lei T , Mao WM , Yang HJ , et al. Study on cancer incidence through the Cancer Registry Program in 11 Cities and Counties, China. Chinese J Epidemiol. 2009;30:1165–1170. 20193588

[5] Olivottu IA , Lesperance ML , Truong PT , et al. Intervals longer than 20 weeks from breast‐conserving surgery to radiation therapy are associated with inferior outcome for women with early‐stage breast cancer who are not receiving chemotherapy. J Clin Oncol. 2009;27:16–23. 19018080 10.1200/JCO.2008.18.1891

[6] Yang B , Wei XD , Zhao YT , Ma CM . Dosimetric evaluation of integrated IMRT treatment of the chest wall and supraclavicular region for breast cancer after modified radical mastectomy. Med Dosim. 2014;39:185–189. 24503002 10.1016/j.meddos.2013.12.008

[7] Kinoshita R , Shimizu S , Taguchi H , et al. Three‐dimensional intrafractional motion of breast during tangential breast irradiation monitored with high‐sampling frequency using a real‐time tumor tracking radiotherapy system. Int J Radiat Oncol Biol Phys. 2008;70:931–934. 18164868 10.1016/j.ijrobp.2007.10.003

[8] Hardee ME , Raza S , Becker SJ , et al. Prone hypo‐fractionated whole‐breast radiotherapy without a boost to the tumor bed: Comparable toxicity of IMRT versus a 3D conformal technique. Int J Radiat Oncol Biol Phys. 2012;82:e415–e423. 22019349 10.1016/j.ijrobp.2011.06.1950

[9] Larry L , Sharpe MB , Frazier RC , et al. Intensity modulation to improve dose uniformity with tangential breast radiotherapy: Initial clinical experience. Int J Radiat Oncol Biol Phys. 2000;48:1559–1568. 11121662 10.1016/s0360-3016(00)01396-1

[10] Rudat V , Alaradi AA , Mohamed A , Ai‐Yahya K , Altuwaijri S . Tangential beam IMRT versus tangential beam 3D‐CRT of the chest wall in postmastectomy breast cancer patients: A dosimetric comparison. Radiat Oncol. 2011;6:1–7. 21418616 10.1186/1748-717X-6-26 PMC3069936

[11] Michalski A , Atyeo J , Cox J , Rinks M , Morgia M , Lamoury G . Dosimetric comparison of 3D‐CRT, IMRT, and static tomotherapy with an SIB for large and small breast volumes. Med Dosim. 2014;39:163–168. 24393498 10.1016/j.meddos.2013.12.003

[12] Ma C , Zhang W , Lu J , et al. Dosimetric comparison and evaluation of three radiotherapy techniques for use after modified radical mastectomy for locally advanced left‐sided breast cancer. Sci Rep. 2015;5:12274. 26194593 10.1038/srep12274 PMC4508617

[13] International Commission on Radiation Units and Measurements: Prescribing, Recording, and Reporting Photon Beam Therapy, Report 50. 1993, *J ICRU*.

[14] International Commission on Radiation Units and Measurements: Prescribing, Recording and Reporting Photon Beam Therapy, Report 62. 1999, *J ICRU*.

[15] Kataria T , Sharma K , Subramani V , Karrthick KP , Bisht SS . Homogeneity Index: An objective tool for assessment of conformal radiation treatments. J Med Phys. 2012;37:207–213. 23293452 10.4103/0971-6203.103606 PMC3532749

[16] Feuvret L , Noël G , Mazeron JJ , Bey P . Conformity index: A review. Int J Radiat Oncol Biol Phys. 2006;64:333–342. 16414369 10.1016/j.ijrobp.2005.09.028

[17] Early Breast Cancer Trialists' Collaborative Group (EBCTCG) , Darby S , McGale P , et al. Effect of radiotherapy after breast‐conserving surgery on 10‐year recurrence and 15‐year breast cancer death: Meta‐analysis of individual patient data for 10801 women in 17 randomised trials. Lancet. 2011;378:1707–1716. 22019144 10.1016/S0140-6736(11)61629-2 PMC3254252

[18] Lewin AA , Derhagopian R , Saigal K , et al. Accelerated partial breast irradiation is safe and effective using intensity‐modulated radiation therapy in selected early‐stage breast cancer. Int J Radiat Oncol Biol Phys. 2012;82:2104–2110. 21640490 10.1016/j.ijrobp.2011.02.024

[19] Freedman GM , Li T , Nicolaou N , Chen Y , Ma CC , Anderson PR . Breast intensity modulated radiation therapy reduces time spent with acute dermatitis for women of all breast sizes during radiation. Int J Radiat Oncol Biol Phys. 2009;74:689–694. 19362779 10.1016/j.ijrobp.2008.08.071 PMC2720600

[20] Van Der Lann HP , Dolsma WV , Maduro JH , Korevaar EW , Hollander M , Langendijk JA . Three dimensional conformal simultaneously integrated boost technique for breast‐conserving radiotherapy. Int J Radiat Oncol Biol Phys. 2007;68:1018–1102. 17379440 10.1016/j.ijrobp.2007.01.037

[21] Freedman GM , Anderson PR , Bleicheer RJ . Five year local control in a phase II study of hypo‐fractionated intensity modula ted radiation therapy with an incorporated boost for early stage breast cancer. Int J Radiat Oncol Biol Phys. 2012;84:888–893. 22580118 10.1016/j.ijrobp.2012.01.091 PMC3419789

[22] Kraus‐Tiefenbacher U , Sfintizky A , Welze G , et al. Factors of influence on acute skin toxicity of breast cancer patients treated with standard three‐dimensional conformal radiotherapy (3D‐CRT) after breast conserving surgery (BCS). Radiat Oncol. 2012;7:217. 23249653 10.1186/1748-717X-7-217 PMC3598440

[23] Sethi RA , No HS , Jozsef G , Ko JP , Formenti SC . Comparison of three‐dimensional versus intensity‐modulated radiotherapy techniques to treat breast and axillary level III and supraclavicular nodes in a prone versus supine position. Radiother Oncol. 2012;102:74–81. 21993404 10.1016/j.radonc.2011.09.008

[24] Recht A , Ancukiewicz M , Alm El‐Din MA , et al. Lung dose volume parameters and the risk of pneumonitis for patients treated with accelerated partial‐breast irradiation using three‐dimensional conformal radiotherapy. J Clin Oncol. 2009;27:3887–3893. 19620489 10.1200/JCO.2008.20.0121

[25] Jo Y , Kay CS , Kim JY , et al. Significance of low‐dose radiation distribution in development of radiation pneumonitis after helical‐tomotherapy‐based hypo‐fractionated radiotherapy for pulmonary metastases. J Radiat Res. 2014;55:105–112. 23757513 10.1093/jrr/rrt080 PMC3885113

[26] Yorke ED , Jackson A , Rosenzweig KE , Braban L , Leibel SA , Ling CC . Correlation of dosimctric factors and radiation pneumonitis for non small cell lung cancer patients in a recently completed dose escalation study. Int J Radiat Oncol Biol Phys. 2005;63:672–682. 15939548 10.1016/j.ijrobp.2005.03.026

[27] Darby SC , Ewertz M , McGale P , et al. Risk of ischemic heart disease in women after radiotherapy for breast cancer. N Engl J Med. 2013;368:987–998. 23484825 10.1056/NEJMoa1209825

[28] Taylor CW , McGale P , Povall JM , et al. Estimating cardiac exposure from breast cancer radiotherapy in clinical practice. Int J Radiat Oncol Biol Phys. 2009;3:1061–1068. 10.1016/j.ijrobp.2008.05.066 18973978

[29] Abo‐Madyan Y , Aziz MH , Aly MMOM , et al. Second cancer risk after 3D‐CRT, IMRT and VMAT for breast cancer. Radiother Oncol. 2014;110:471–476. 24444525 10.1016/j.radonc.2013.12.002

[30] Grantzau T , Mellemkjaer L , Overgaard J . Second primary cancers after adjuvant radiotherapy in early breast cancer patients: A national population based study under the Danish Breast Cancer Cooperative Group(DBCG). Radiother Oncol. 2013;106:42–49. 23395067 10.1016/j.radonc.2013.01.002

[31] Gao X , Fisher SG , Emami B . Risk of second primary cancer in the contra lateral breast in women treated for early‐stage breast cancer: A population‐based study. Int J Radiat Oncol Biol Phys. 2003;56:1038–1045. 12829139 10.1016/s0360-3016(03)00203-7

[32] Di Betta E , Fariselli L , Bergantin A , et al. Evaluation of the peripheral dose in stereotactic radiotherapy and radio‐surgery treatments. Med Phys. 2010;37:3587–3594. 20831066 10.1118/1.3447724 PMC2902541

[33] Kaufman EL , Jacobson JS , Hershman DL , Desai M , Neugut AI . Effect of breast cancer radiotherapy and cigarette smoking on risk of second primary lung cancer. J Clin Oncol. 2008;26:392–398. 18202415 10.1200/JCO.2007.13.3033

[34] Chopra S , Dinshaw KA , Kamble R , Sarin R . Breast movement during normal and deep breathing respiratory training and set up errors: Implications for external beam partial breast irradiation. Br J Radiol. 2006;79:766–773. 16940376 10.1259/bjr/98024704

[35] Remouchamps VM , Letts N , Yan D , et al. Three‐dimensional evaluation of intra‐ and inter‐fraction immobilization of lung and chest wall using active breathing control: A reproducibility study with breast cancer patients. Int J Radiat Oncol Biol Phys. 2003;57:968–978. 14575827 10.1016/s0360-3016(03)00710-7

[36] Gagel B , Demirel C , Kientopf A , et al. Active breathing control (ABC): Determination and reduction of breathing‐induced organ motion in the chest. Int J Radiat Oneol Biol Phys. 2007;67:742–749. 10.1016/j.ijrobp.2006.09.052 17197133

